# Student and supervisor perspectives on the effectiveness of community-based placements for occupational therapy students

**DOI:** 10.1186/s12909-021-02492-3

**Published:** 2021-01-25

**Authors:** Anat Golos, Esti Tekuzener

**Affiliations:** School of Occupational Therapy, Faculty of Medicine, the Hebrew University, Mt. Scopus, P.O.B: 24026, 91240 Jerusalem, Israel

## Background

Practice placement education in occupational therapy is a fundamental component in the development of students’ professional identity [1], and it enables opportunities for practice and application of theoretical knowledge with clients across the lifespan in a variety of settings [2, 3]. The literature suggests the need for extensive research on practice placement education experiences [2], as well as the expansion of types of practice placements [4, 5], and likewise supports the successful influence of these experiences on students’ professional outcomes [6, 7]. However, there is limited research focused on students’ perspectives, including their expectations and satisfaction regarding practice placement. Even fewer studies exist on the perceived benefits of the different practice placements prior to and post placement [5, 8]. Expectations are defined as a person’s beliefs that a certain behavior or outcome will occur as a result of a specific event; satisfaction is defined as an emotional response to the gap between expectations or desires and the perception of what one actually received or gained [9].

Occupational therapy clinical placement models can be classified as either role-established, a more traditional placement; or role-emerging, a nontraditional placement. The role-established placement model occurs in various recognized and approved clinical settings where occupational therapists work, such as medical and educational institutions, as well as community services with a broad range of health-related services [10–12]. The role-emerging practice model focuses on developing occupational therapy roles in settings that lack an established occupational therapist, to enable individual, group, and community participation in occupations [5, 13, 14]. In these settings, the occupational therapy is not routinely provided, but the potential exists [15]. In a role-emerging practice placement, an on-site professional (not an occupational therapist) provides students with day-to-day supervision in the settings, while formal professional supervision is provided weekly by an off-site occupational therapist [2, 5, 16].

Using a role-emerging practice model corresponds with the belief that skills and training can and should be used to develop and expand established, emerging, and re-emerging areas of practice [17]. Using a community-based practice model enables the development of new emerging practices within the community [2, 18, 19]. In emerging areas of practice, occupational therapists meet the growing needs by using their unique skills and training to more specifically serve individuals in the environment in which they live [17]. In occupational therapy, a community can be defined as a group of people engaged in collective occupations, with a collective sense of meaning. Occupations refer to the everyday activities that people do as individuals, in families, and with communities, to occupy time and bring meaning and purpose to life [20]. Understanding environments and contexts where occupations can and do occur provides practitioners with insights and influence on engagement [18]. Community can also be defined as a setting in which people reside, build relationships, and engage in health practices [18, 21, 22]. Community-based occupational therapy practice encompasses a wide variety of practice options that arise from the needs of specific populations, such as individuals or groups of people who share public services and have a common interest. Using a community-based role-emerging model is needed in order to continue the development of new areas of practice, or to practice in settings where the occupational therapist re-establishes services in a new and innovative way [5, 13].

In alignment with the health-care emphasis on promoting health and wellness, occupational therapy students are encouraged to develop the knowledge and skills necessary to work in the community-based role-emerging settings. In order to maintain their competency in serving new populations, environments, and systems, students need to be exposed to role-emerging areas during practice placements [16, 17]. This may better prepare students to evolve into more diverse and holistic therapists in settings where the role of occupational therapy is undefined [23]. Role-emerging placements may have advantages, since they can enrich students’ professional and personal growth and development, and allow students to explore, establish, and expand roles in the community [17, 24, 25]. This may be an opportunity where students can apply academic coursework and resources to address challenges facing communities, such as community needs, health-care disparities, and civic engagement [26]. Role-emerging placements have been found to enhance students’ independence, clinical reasoning, problem-solving and communication skills, as well as broaden their autonomy, self-directed learning, and ability to advocate [3, 4, 13, 17, 23].

Limitations of role-emerging placements do exist. Students and supervisors report a lack of appreciation for the role of occupational therapy, difficulty in developing a professional identity, a lack of sufficient client contact in certain settings, and a disadvantage for weaker students in such placements [16, 24, 27]. Clarke (2014) [4] noted practical difficulties linked to a lack of communication between on-site and off-site supervisors, discrepancy in staff attitudes, supervisors who were only part-time, and different expectations of students and university staff. The role-emerging placements can also include projects that are developed to address specific needs, identified in collaboration with the agency in the placement setting. During project implementation, students are self-directed and independent [5, 11].

Current research has examined the experiences of occupational therapy students participating in community role-emerging practice placements, mostly by using quantitative methods [19, 23, 28]. Few studies have been conducted during the placements [28], prior to, and/or upon completion of placements [8]. Students’ experiences in role-emerging practice placements need to be continually evaluated in order to facilitate their learning process [28], and to ensure that those experiences enable them to develop the necessary professional and personal knowledge and skills, as well as to understand their potential contribution to the community.

Due to the need for continually optimising the experiences within the framework of the students’ placements, and the need for both quantitative and pre-post qualitative information regarding the placements process, the purpose of this study was to evaluate the impact of the experience of participating in community-based role-emerging practice placements from the perspectives of undergraduate occupational therapy students and supervisors. Specifically, in the first stage of research, we aimed to draw upon students’ experiences in community-based role-emerging practice placement, using focus groups to collect qualitative data. In the second stage of research, based on the information obtained from the focus groups, we aimed to examine: (1) students’ perceptions prior to and after completion of role-emerging practice placements in four categories: setting and supervision, personal skills, professional skills, and community; (2) the differences in students’ perceptions regarding their experiences in full-time vs. part-time placements; and (3) supervisors’ experiences in guiding students in the role-emerging practice placements, shared after completion.

## Method

### Research design

This study was conducted among undergraduate occupational therapy students, who participated in community-based role-emerging practice placements during their second to fourth years of academic studies. We used a mixed method design that included focus-group interviews, conducted as the first stage of research [29], along with pre-post questionnaires at the second stage of research. Additional initial information was gathered from supervisors using a short online questionnaire.

The University Ethics Committee (institutional review board) approved the study. Consent was obtained in writing from students, and online from supervisors. Participants were informed that they could withdraw from the research at any time without giving reasons, and that withdrawal would not affect their studies or employment.

### Participants

Participants included 41 undergraduate occupational therapy students, from the School of Occupational Therapy of the [Institutional details], who participated in community-based role-emerging practice placements during 3 years of academic studies (2016–2018). Seven professional occupational-therapist supervisors were also included.

Community-based role-emerging practices are integrated into the framework of occupational therapy students’ placements. These students are mandated to complete a minimum of a thousand hours of clinical placements [30]. The occupational therapy program is a three-and-a-half-year undergraduate program. As part of the educational curriculum, students completed three practice placements consecutively in the second, third, and fourth years of the program, for a duration of 8–12 weeks. Students were assigned (independently or in pairs) to role-emerging practice placements only if they expressed a desire to be placed in these settings. Role-emerging placements were classified as either full-time or part-time. Students in full-time placements were present for a five-day work week. Students who participated in part-time placement were present once a week, and spent the remainder of their week in a setting with established occupational therapy service.

The community-based practice placements that were selected were not defined as medical or therapeutic settings (i.e., kindergartens, community mental health centers, daily community senior centers, and academic support centers; see Appendix**)**, and they had no on-site occupational therapy services. The populations in the selected settings were defined as at risk, and/or populations that could derive benefit from occupational therapy intervention which focused on improving their participation in daily activities, as well as their health and wellbeing (i.e., children of foreign workers and asylum seekers, neighborhood senior citizens, people with mental health disorders, and students with various medical conditions; see Appendix 1).

In addressing the known limitations of the role-emerging practice placement, the school coordinator recruited professional off-site occupational therapist supervisors with a minimum of 5 years of experience. Supervisors visited the settings before the practice placements began and met the population and staff of the setting, in order to explain the contribution of occupational therapy and define the role-emerging practice placement. They then designed a program for each practice placement according to the role-emerging type, the practice duration (8–12 weeks), the placement setting (e.g., day center, kindergarten), and the population involved. During the practice placement, the off-site supervisors met the students in their settings (twice a week or more for full-time, and less for part-time) and served as role models. In addition, they were available for students via email or telephone as necessary throughout the week. As part of their practice placement education, students were required to carry out academic assignments, such as planning and implementing intervention processes for the population in the placements. In addition to ongoing feedback from their supervisors and the school coordinator, students were scored using a university-built final assessment of practice placement, which evaluated their clinical performance and completion of academic requirements.

### Procedure

In the first stage of research, two focus-group interviews (qualitative data) were conducted with 14 students who had participated in community-based role-emerging practice placements during one academic year, in order to learn about their experiences. The focus groups were moderated by the two authors and were conducted as recommended by Bryman (2015) [31], and Krueger and Casey (2008) [32]. Throughout the focus group meetings, open-ended questions were presented (e.g., “What were the positive and negative aspects of the experience?”; “Did the supervision and placement address your needs?”; “Did the experience contribute to the development of your skills?”; “Is there any recommendation you would suggest for change?”). In addition, moderators asked the students questions in order to fully understand their experience (“Do you have anything else to add?”; “Have we missed anything?”). The interviews, which took place in a private room at the school of occupational therapy, were recorded and transcribed by the first author and another team member.

In the second stage of the research, some changes were implemented during two academic years, based on the information obtained from the focus groups (e.g., revising goals, and improving coordination with supervisors and professional staff). Since no appropriate measurement tools were identified in order to evaluate students’ perceptions regarding their practice placement, a new tool needed to be created. The focus groups allowed us to refine and rate the points of reference significant for students’ placements, which can be used among a large group of students. Therefore, pre-post questionnaires were developed as a part of a larger body of research [33] in order to re-evaluate the practice placement experience. Twenty-seven additional students from the following two academic years completed the questionnaires (quantitative data) 1 week before and 1 week after their role-emerging practice placements (these students’ placements matched those of students from the focus groups in the previous year). In addition, the supervisors were asked to complete a short online questionnaire after the completion of placements.

### Measure

In the first stage of research, qualitative information through focus groups was collected from students using open-ended questions (as mentioned in the Procedure section). Questions included student experiences in their practice placement, levels of satisfaction, and recommendations relating to their skills, the supervision, and the settings. In the second stage of research, the quantitative information was collected from the students’ questionnaires.

#### Students’ questionnaires

Student’s perceptions, expectations, and satisfaction levels were evaluated via the questionnaire items, which were based on the literature, clinical experience, and reviews from faculty, supervisors, and students. Two versions of the questionnaire were designed: one for use prior to placements, and another upon completion of placements. Each version of the questionnaire included 29 items, using a 5-point Likert scale (1 = “not at all”; 5 = “extremely”). The items were divided into four distinct categories: (1) practice placement and supervision (10 items); (2) personal skills (9 items); (3) professional skills (6 items); and (4) contribution to the community (4 items). The questionnaire categories and items are presented in Table 1. The pre-placement questionnaire was designed to explore the student’s perceptions and expectations prior to placements, based on their personal preferences and their previous experiences in practice placements. The items were written as outcome measures in order to predict future behaviors (e.g., *“I expect that the supervisor will provide individualised, direct, specific supervision”*); they also evaluated students’ perceptions about their personal and professional skills (e.g., *“I think I have enough theoretical knowledge to succeed in the placement experience”*). The post-placement questionnaire included items that evaluated the change in the predicted behaviors during the placement experience (e.g., *“The supervision I received during the placement experience enhanced my professional knowledge and skills”*), as well as students’ perceptions regarding the contribution and importance of occupational therapy services to the community (e.g., *“I think that my work during the placement contributed to the understanding of occupational therapy services in the community”*). Evaluation of the questionnaires’ initial psychometric properties as a part of a larger body of research included internal consistency, which was found to be adequate for all four questionnaire categories (Cronbach’s α = 0.763, 0.840, 0.793, 0.721; respectively) [33].

Additional qualitative information was collected from supervisors using a short online questionnaire developed for this study, in order to collect data from the on-site supervisors regarding their experience during the role-emerging practice placements. This questionnaire, presented in Table 2, includes seven items (e.g., *“The supervision I gave to the students helped them to plan and implement intervention”; “I was able to provide students with appropriate feedback”*) which use a 5-point Likert scale (1 = “strongly disagree”; 5 = “strongly agree”).

### Data analysis

Overall data collection involved both qualitative and quantitative data, a combination which is effectively used in health science [34], as well as for evaluating service programs [35]. Qualitative information provided by students from the focus-group interviews was recorded, transcribed, discussed by the authors with an additional team member, and coded into main themes according to conventional content analysis [36]. Statistical analyses of quantitative data from both students’ and supervisors’ perspectives were conducted using the Statistical Package for the Social Sciences (SPSS-Version 25). Descriptive statistics were used. The significance level was set at 0.05. Due to small sample sizes, as well as abnormal distribution, nonparametric tests were used. Wilcoxon tests were used in order to examine the changes in scores from pre to post in each questionnaire’s category and items. Additionally, based on nonsignificant (*p* > 0.05) differences that were found between the pre-scores of the two types of placements (full-time vs. part-time), Mann-Whitney tests were used to compare the total post-scores of these types in each category.

## Results

### Students’ perspectives regarding their experience: qualitative information

Based on the data collected from the focus-group interviews, three school practice-placement team members (the two authors and a supervisor) observed the following three main themes according to conventional content analysis [36]: (a) Environmental characteristics of the practice placements (settings); (b) Supervision model and supervisor; and (c) Students’ professional and personal skills.

#### Environmental characteristics of the practice placements (settings)

Relating to the physical environment, in some of the practice placements no separate spaces or specific equipment (such as assessment tools and rehabilitation aids) were used for individual or small-group evaluations, or for the intervention process, as in some medical or rehabilitation placements. Therefore, some students felt they lacked access to equipment and a separate space needed for evaluation and intervention. For example, Interviewee #01 noted: *“There was no evaluation equipment in the setting, so we needed to achieve assessment tools, as well as to improvise and adapt equipment.”* Interviewee #02 noted: *“I needed to manage with the existing space in order to evaluate the level of performance and abilities of some individuals in the setting.*”

The social environment in practice placements included the staff members and the population. Some students reported that they expected the staff members to be more prepared for the practice placement, as well as to be more aware of their learning process and role as students. However, they felt that these expectations did not match those of the staff members, which expected students to start their intervention from earlier stages in the practice placement. As Interviewees #03 and 04 noted: *“It took time to understand what was expected of us…. It seems that the school coordinator needed more coordination with the staff in order to better explain our learning process.”* Additionally, all interviewees expressed satisfaction with the support from and collaboration with the staff members. For example*,* Interviewee #5 noted: *“We received guidance, explanations, and support from the staff members that enabled us to take the initiative and be independent.”* Relating to the population in the placements, some students reported that the intervention goals, which were included in the program design, were general and not specific to the population. As a result, they needed more time for additional evaluation and identification of the population’s needs by using observation and standardised evaluation tools. They also needed more time for building interpersonal relationships and for planning specific intervention activities. As Interviewees #6 and 7 noted: *“Although there was a general plan, we still needed more time for connecting with the population in the setting, planning evaluations and activities for intervention for specific individuals and groups, as well as for documentation and reporting the process and results…. I felt that I could understand the main goals, but I needed the plan to be clearer, because it took us time to know and define the specific needs of the population.”*

#### Supervision model and supervisor

Students mentioned the need to adapt the supervision model to the practice placement and to their needs (such as adapting individual and/or group supervision to the supervisor’s availability). They all mentioned that the off-site supervisor provided them with personal and professional support, answered their questions, directed them to learning resources, made connections between theory and practice, was empathetic and sensitive to their needs, and served as a role model. As Interviewees #8, 9, and 10 noted: *“The supervision was effective and contributed to my knowledge about the specific needs of the population in the setting. I learned how to connect with the population and define their needs…. I had the opportunity to observe my supervisor in a few evaluation sessions, while she encouraged me to ask questions and think about possible explanations for the functional status of the person. For me, the supervisor served as a role model of an empathic and professional occupational therapist…. It was good and challenging, thanks to the supervisor, who provided me with professional knowledge, answered my questions, and encouraged me to better understand community involvement. My supervisor also expressed a positive attitude and support.”*

#### Developing professional and personal skills

During the role-emerging practice placements, students reported that they needed to be more creative, flexible, independent learners, and to take the initiative to solve problems independently, since on-site occupational therapy services were not available in the practice placements. This was especially mentioned by a majority of students who executed projects during their practice placement. All students reported that they were required to display communication skills and collaborate in relationships with different staff members who were not occupational therapists. As Interviewees #10 and 11 noted: *“The role-emerging practice placement required me to initiate and plan group sessions and be more independent, compared to my previous practice placement, since no other occupational therapists were involved…. It required creative thinking with a lot of flexibility in order to implement the intervention during the routine of the population in the setting.”* Students who were assigned in pairs to the same placement felt that this arrangement was supportive and enabled them to better experience and develop their skills, since they could collaborate in acquiring knowledge and improving their clinical reasoning, as well as support each other in implementation of the intervention. As Interviewees #13 and 14 noted: *“It was very useful for us to be a pair, as we had a lot of work and we needed to initiate and be active… I felt that being together with another student promoted my skills and also contributed to my professional self-competence.”*

### Students’ perspectives regarding their experiences: pre-post questionnaires

Students’ perspectives regarding their experiences in role-emerging practice placements were evaluated using the above-described questionnaires, which were completed prior to and upon completion of their placements. As seen in Table 1, the results of the Wilcoxon tests indicated that in the category of *practice placement and supervision,* the total pre-scores were significantly higher than the post-scores (z = − 3.557, *p* < .001). Similarly, in all items there were significant decreases in scores from pre to post, except for a nonsignificant increase in *interpersonal and professional support from staff members*.

In the category of *personal skills*, the total post-scores were found to be significantly higher than the pre-scores (z = − 2.805, *p* < .01). Overall, most items in this category increased from pre to post, with four items showing significance. In the category of *professional skills*, total post-scores were also significantly higher than the pre-scores (z = − 3.152, p < .01). In all items of that category, increases were evident in scores from pre to post, with significance in two items.

Finally, in the category of *community,* the total pre-scores were higher than the post-scores, but differences were not significant (z = 0.881, *p* > .05). In most items in this category, there was also a nonsignificant decrease in scores from pre to post. However, a nonsignificant increase was found in the item of *commitment to contribute to the community* (Table 1).

**Table 1:** Students’ perspectives regarding community-based role-emerging practice placements before and after completion of placements

Categories and items	Pre-test		Post-test		Z
	M	SD	M	SD	
**Practice placement and supervision** (10 items)					
1. Orientation process in the setting	4.41	0.69	3.70	0.95	−2.758^**^
2. Matched expectations with supervisor	4.82	0.48	3.85	1.10	−3.141^**^
3. Expectations of practice placement	4.78	0.51	3.48	1.10	−3.890^***^
4. Adequate amount and type of supervision	4.37	0.74	3.35	1.37	−2.810^**^
5. Information about setting policies and procedures	4.48	0.64	3.42	0.90	−3.452^**^
6. Supervisor provides individualized, direct, specific supervision	4.67	0.56	3.98	0.96	−2.686^**^
7. Setting supports academic learning	4.11	0.80	2.67	0.92	−3.789^***^
8. Supervisor competency in professional knowledge and skills	4.74	0.45	3.69	1.07	−3.357^**^
9. Interpersonal and professional support from staff members	3.56	1.01	3.96	0.98	−1.421
10. Staff preparation	3.96	0.85	3.36	1.08	−2.452^*^
**Total**	**4.39**	**0.39**	**3.55**	**0.69**	−3.557^***^
**Personal abilities** (9 items)					
1. Well-integrated into practice placement	4.07	0.47	4.52	0.70	−2.556^*^
2. Collaborative relationship with staff and population	4.33	0.48	4.72	0.49	−2.500^*^
3. Collaborative relationship with the supervisor	4.52	0.57	4.26	0.72	−1.147
4. Ability to receive constructive feedback	4.41	0.50	4.31	0.47	−1.000
5. Opportunities to work creatively	4.22	0.64	4.26	0.71	−0.277
6. Opportunities to work independently	3.69	0.84	4.70	0.47	−4.014^***^
7. Opportunities to initiate new/ innovative ideas	3.96	0.59	4.37	0.57	−2.668^**^
8. Ability to problem-solve through clinical reasoning	4.15	0.53	4.26	0.59	−0.905
9. Ability to ask questions	4.19	0.68	4.26	0.59	−0.486
**Total**	**4.18**	**0.41**	**4.41**	**0.36**	**2.805-** ^**^
**Professional abilities** (6 items)					
1. Competency in theoretical knowledge	3.30	0.72	3.43	0.74	−0.698
2. Competency in evaluation process	3.74	0.76	4.06	0.68	−1.430
3. Competency in intervention process	3.70	0.65	4.41	0.57	−3.597^***^
4. Collaborates with others (colleagues, family/ support system, and other staff)	3.30	0.67	3.48	0.90	−1.231
5. Professional contribution to the practice placement	4.07	0.68	4.59	0.50	−2.488^*^
6. Articulates and implements OT role	3.78	0.80	4.07	0.92	−1.311
**Total**	**3.65**	**0.51**	**4.03**	**340.**	−3.152^**^
**Community** (4 items)					
1. Contribution of knowledge and experience to the community	4.33	0.69	4.20	0.44	−1.037
2. Enhances the understanding of OT’s role in the community	4.56	0.64	4.27	0.78	−1.507
3. Commitment to contribute to the community	4.07	0.92	4.36	0.57	−1.328
4. Partnering in developing community-based OT services	4.00	0.90	3.83	0.87	−1.224
**Total**	**4.24**	**0.59**	**4.18**	**0.43**	**−0.881**

Notes: *OT* Occupational therapy; *N* = 27; Z = Wilcoxon tests, ^*^p **≤** 0.05; ^**^p **≥** 0.01; ^***^p < 0.001

### Students’ experiences in full-time vs. part-time practice placements

Based on nonsignificant (*p* > 0.05) differences between the pre-scores, Mann-Whitney tests were used in order to compare the post-scores of the full-time and part-time placements. The results indicated nonsignificant (p > 0.05) differences in total post-scores between full-time and part-time placements in all categories, except for close-to-significant differences (Z = − 1.923, *p* = 0.055) in the *personal skills* category, where we saw higher scores for full-time placements (*n* = 18, Mean = 4.50, SD = 0.31, Median = 4.44) compared to part-time placements (*n* = 9, Mean = 4.21, SD = 0.39, Median = 4.22).

### Supervisors’ perspectives regarding their experiences

Seven supervisors reported their experiences with students during the role-emerging practice placements through a short online questionnaire. As seen in Table 2, most supervisors rated all statements as “agree” or “strongly agree”. Most supervisors reported that the supervision experience was overall positive, and all of them reported that the role-emerging practice placement was productive and contributed to the setting. Additionally, most supervisors reported that they were able to assess students’ performance, provide feedback, and assist in planning and implementing interventions. They also reported that they were sufficiently prepared for the supervisory role, and most indicated that supervision of two students in the same setting was advantageous. Although most supervisors expressed positive feedback, two of them were less positive. This should be investigated in further studies that include structured interviews and additional supervisors.

**Table 2:** Supervisors’ perspectives regarding their experience in role-emerging practice placement (*n* = 7)

Statement	Strongly disagree % (n)	Disagree % (n)	Undecided % (n)	Agree % (n)	Strongly agree % (n)
1. The supervision I gave to the students helped them to plan and implement intervention				28.6 (2)	71.4 (5)
2. I was able to assess students’ functioning adequately		14.3 (1)	14.3 (1)	57.1 (4)	14.3 (1)
3. I was able to provide students with appropriate feedback		14.3 (1)	14.3 (1)	57.1 (4)	14.3 (1)
4. The supervision of two students in the same setting was an advantage			14.3 (1)	14.3 (1)	71.4 (5)
5. I felt sufficiently prepared for supervising the students		14.3 (1)		85.7 (6)	
6. Overall, my experience in supervising students was positive			14.3 (1)	28.6 (2)	57.1 (4)
7. The practice placements were productive and contribute to the setting.				14.3 (1)	85.7 (6)

## Discussion

This study evaluated the experiences of undergraduate occupational therapy students who participated in community-based role-emerging practice placements, along with their supervisors’ perspectives. The placements occurred in community settings that were not defined as therapeutic and had no occupational therapy services. The population was defined as at risk and/or one which may benefit from occupational therapy intervention. The intervention sought to meet the community’s desired outcome [18, 37], and to enable individuals to participate in daily life routines within their environments.

Data collection involved both qualitative and quantitative data, using focus groups in the first stage of research, as well as pre-post questionnaires and a short online questionnaire in the second stage of research. These enabled us to gain a broad perspective on the experience of community-based role-emerging practice placements. The data will be discussed under three main themes related to the community-based role-emerging practice placements: (a) practice placements (settings) and supervision; (b) students’ professional and personal skills; and (c) contribution to the community.

Regarding the *practice placements (settings) and supervision*, the overall results showed that students were less satisfied by the settings and supervision when compared to their expectations prior to placement. The gaps between students’ expectations and their level of satisfaction can be explained by the fact that no on-site occupational therapy services were available in settings. Although supervisors oriented and prepared staff members for the practice placements, some placements had limited previous experience in supervising students. Additionally, some staff members were less aware of students’ needs and did not provide proper orientation for the settings; moreover, no discussion of comparative expectations took place at the beginning of the practice placement. In addition, since the off-site supervisor was not present in the daily routine, the staff members expected the students to start intervention from earlier stages in the practice placement, while the students expected to have more time for their learning process, getting to know the population and defining their needs. The only increase in score was with regard to the support from staff members, although it was not significant. This increase was supported by students’ reports during the focus groups, indicating sufficient support from staff members in the settings. Regarding the supervision, it seems that although supervision was implemented and the supervisors were available to students during their practice placement period, some students still expressed less satisfaction with off-site supervision when compared to their expectations prior to placement.

It should be noted that students generally seemed to have high prior expectations from the settings and supervision. These results support a previous study, which indicated that educators and students who participated in focus groups and interviews, in order to address their perspectives regarding quality practice placement experiences, reported on the need for clear and high expectations from both the placements and supervisors [8]. However, unlike our study, that study used only a qualitative evaluation method, and it did not compare the students’ expectations with their satisfaction levels upon completion of their placements. In our study, despite the decreased score, students’ satisfaction levels were still reported as high. These high satisfaction levels support an additional study that assessed overall perceptions and satisfaction among 67 students with their fieldwork, and indicated a high satisfaction level mainly with the supervisors rather than the settings [38]. However, unlike our study, this study included only a four-question assessment, which was sent to students 1 month before the completion of their fieldwork.

Students’ levels of satisfaction with the setting and supervision were also reported through the focus groups, and they seemed to vary. Overall, students reported positive impressions of their off-site supervisors, who provided them with support, directed them to learning resources, were empathetic and sensitive to their needs, and served as role models. This result is supported by a previous study indicating students’ perceptions of the supervisory relationship as supportive [6]. Similar positive experiences were reported by most supervisors, indicating that they were able to provide feedback and assist the students in planning and implementing interventions. However, students noted the need to adapt the supervision model to the setting and to their needs, as well as to better customize the program to the population in the setting. Students also noted a lack of resources for intervention, and the need for better preparation of the staff members in order to have more realistic expectations from them as students. These results support other studies that highlighted concerns and challenges of the role-emerging model relating to the setting and supervision, which may result in false expectations by students, and therefore require structured guidance [28, 39]. Since students are expected to adapt to the existing setting and to be able to learn by using supervision [10, 40], their expectations and needs should be taken into account when preparing them for community-based role-emerging placements. The varied responses collected during the study regarding the setting and supervision confirm the need for further research that will include both quantitative and qualitative data from different perspectives, as well as from additional participants. Using both quantitative and qualitative methods can also strengthen the findings [34].

With reference to students’ *personal and professional skills*, the quantitative data indicated an overall significant increase in total scores, showing that students reported improvement in both their professional and personal skills following their experience in these placements. Increases in scores were also noted in all items (although not all were significant), except for a nonsignificant decrease in two professional-skills items: *collaborative relationship* and *receiving feedback from the supervisor*. This may be explained by the fact that both items are related to the individual student’s relationship with the supervisor. This corresponds with the findings that students were less satisfied by the supervision, compared to their prior expectations. The improvement in students’ perceptions regarding their skills was also noted during the focus groups, where students reported that the experience enabled them to be more creative, flexible, independent learners, and to solve problems with independent initiative, as well as to communicate and collaborate with others. These results support previous studies indicating that role-emerging practice placements contribute to the student’s personal and professional abilities [13, 17, 24, 28, 41], and support the belief that the role-emerging model can also be used as an appropriate model of practice placement [28]. An additional and interesting finding was the close-to-significant higher score in personal skills after completion among students who participated in full-time placements, compared to those finishing part-time placements. This may be further investigated in order to evaluate the potential contribution of a full-time experience specifically to students’ personal skills. Future research is recommended to better identify the benefits of each placement type.

Relating to *community*, the overall nonsignificant decrease in scores following placements showed that students did not report improvement in their perceptions of the importance of community engagement or their contribution to the community. The only increased score was regarding their *commitment to contribute to the community*, although this was not significant. It should be noted that similar to students’ prior expectations regarding the settings and supervision, their perceptions regarding the community were also high prior to placement. Based on previous studies indicating that role-emerging placements allow students to explore, establish, and expand occupational therapy roles in the community, and to develop their own professional role [16, 17, 24, 25], we assumed that students who participated in such placements would improve their perceptions regarding the overall role of occupational therapy and its contribution to the community, especially in settings with no on-site occupational therapy services. The results did not support this assumption, which can be explained by the fact that each student experienced only one specific community-based setting. Therefore, each student experience was limited to the particular setting and did not offer a wide perspective regarding the broader contribution of occupational therapy to a community in general. It can be assumed that more opportunities can enable students to address the challenges facing a community [23], which is necessary for supporting the belief that the role-emerging model may contribute to the need for developing and expanding areas of practice in a community [42]. These perceptions regarding the importance of community engagement, and the contribution of occupational therapy to a community, need to be further investigated among a larger sample of students.

### Clinical implications

Educators and practice placement coordinators need to continue developing community-based role-emerging practice placements. This will require them to identify appropriate placements and supervisors, and collaborate with staff members throughout the process, while continuing to evaluate the services. Supervisors need to be aware of a student’s expectations and needs before and during the practice placements. They also need to better strengthen collaboration and relationships with students, in order to provide them with ongoing guidance and feedback related to their learning process. Better preparation should also be developed with the staff members, in order for them to better understand the potential contribution of occupational therapy to the placements. Additionally, the staff members need to be aware of the importance of preparing placements and implementing appropriate orientation for students, as well as being aware of students’ learning processes and needs. Supervisors also need to support and encourage students by increasing their awareness and knowledge regarding the importance of community engagement and the contribution of occupational therapy in developing community partnerships. This can be enhanced by exposing students to additional community-based placements. Furthermore, due attention should be given to the students’ and supervisors’ feedback indicating an advantage in assigning two students to the same practice placement, which allows them to collaborate and support each other. The authors strongly recommend taking this into consideration when assigning students to community-based role-emerging practice placements.

### Limitation and future studies

In interpreting this study’s findings, several limitations should be taken into account. First, the data included a small sample of students, with unequal representation of full-time vs. part-time placements, due to insufficient resources allocated to role-emerging placements. Future studies should examine greater numbers of students and supervisors who are involved in such practice placements, while also expanding the comparisons between role-emerging types. Larger samples will allow investigation of additional psychometric properties of the students’ questionnaires in future studies. It is important to note that students’ satisfaction levels with their placements may be influenced by the fact that these are authentic real-life situations. Further research is needed to explore this possibility. In addition, the supervisor questionnaire used for the purpose of this study was a self-reported tool reflecting mainly positive feedback. It should be noted that in addition to this questionnaire, each supervisor completed final evaluations using a template to assess each student’s clinical performance and process. Supervisors’ feedback should be evaluated in further studies using quantitative and qualitative methods. Finally, this study described only students’ and supervisors’ perspectives; future studies should also include data from the perspective of the populations being served, as well as from other community partnerships, using both qualitative and quantitative methods. Examining the long-term effectiveness of community-based role-emerging services, including cost-efficiency, is also needed.

## Conclusions

This study examined students’ experiences prior to and upon completion of their community-based role-emerging practice placements, including aspects of contribution to the community, by using both quantitative and qualitative data. Our results support the belief that community-based role-emerging practice placements contribute to the professional and personal development of occupational therapy students, and to a high rate of overall student satisfaction. The study supports the positive aspects and challenges of these placements, mainly from the students’ perspective, and also from their supervisors’ perspective. This study highlights the need for further research in order to better evaluate the quality of community-based role-emerging practice placements from different perspectives and among larger groups of participants, to compare different types of placements, and to evaluate the benefits of placing students in small groups or pairs. This may contribute to the development and adaptation of community-based practice placements to fit the specific placement and population needs.

Role-emerging models can be also appropriate for use as part of a student’s practice placement education, with the potential to contribute to developing and expanding areas of practice in the community. Due to the necessity and value of community-based role-emerging practice placements, better compatibility is required in matching students’ expectations with those of community partnerships and supervisors. This may promote students’ positive experiences, enhance their professional and personal development, and further the development of the occupational therapy profession. Role-emerging practice placements can also serve as a valuable contribution to professionals and stakeholders within communities, who do not as yet benefit from occupational therapy services.

## Data Availability

The dataset generated and analysed during the current study are available to the authors, but are not publicly available due to ethical guidelines.

## Appendix

### Examples of community-based role-emerging practice placements

**Table:** Table 3

Setting	Population	Areas of focus in program design
Kindergarten for children of foreign workers and asylum seekers	Children (3–6 years) of foreign workers and asylum seekers, who are at risk for educational delays due to low socio-economic status, as well as lack of regular access to health and welfare services.	Evaluate children’s performance of school-related tasks; provide strategies to facilitate their full participation in daily routines; Plan developmentally appropriate instructional activities (social skills, motor coordination, self-regulation skills, problem-solving abilities); Guide educational staff.
Senior community activity center	Neighborhood senior citizens aged 60+, participating in socialization groups for occupational, intellectual, and recreational enrichment.	Evaluate seniors’ needs and interests for meaningful activities; Create and implement health programs to assist greater social participation; Provide information about community mobility, environmental modifications to increase ease, safety, and independence with daily tasks; Guide center staff.
Community *center* for people living with *mental* illness	People living with mental illness, participating daily in a community group environment where they can socialize, participate in recreational activities, and learn new skills.	Work with members and staff to manage all operations: clerical, food services, employment programs, housing support and placement, benefit advocacy, case management, financial planning, social programs, and continuing education support.
Academic support Center	Students with various medical conditions (such as learning disabilities, attention deficit disorders, motor and sensory disabilities).	Assist students in developing and improving academic skills (ergonomics, computer use, time management, study skills); Plan to meet different issues (rights, treatment referrals, social participation); Raise staff awareness on issues such as adult sensory regulation, health promotion, occupational balance, and accessibility.
