# Correction to: Student and supervisor perspectives on the effectiveness of community-based placements for occupational therapy students

**DOI:** 10.1186/s12909-021-02528-8

**Published:** 2021-02-15

**Authors:** Anat Golos, Esti Tekuzener

**Affiliations:** School of Occupational Therapy, Faculty of Medicine, the Hebrew University, Mt. Scopus, P.O.B: 24026, 91240 Jerusalem, Israel

**Correction to: BMC Med Educ 21, 73 (2021)**

**https://doi.org/10.1186/s12909-021-02492-3**

Following publication of the original article [1], the authors notified us that the Abstract is missing.

The original article has been corrected.
